# Clonorchiasis sinensis detected by laparoscopic exploration of biliary tracts in two patients with obstructive jaundice

**DOI:** 10.1186/s12879-019-3679-y

**Published:** 2019-01-08

**Authors:** Xialei Liu, Genglong Zhu, Chaonong Cai, Zhiyue Lv, Jian Li

**Affiliations:** 1 Department of Hepatobiliary, the Fifth Affiliated Hospital of Sun Yat-sen University, Zhuhai, 519000 China; 2 Zhongshan School of Medicine, Sun Yat-sen University, Guangzhou, 510080 China; 3 Key Laboratory of Tropical Disease Control (Sun Yat-sen University), Ministry of Education, Guangzhou, 510080 China; 4 Provincial Engineering Technology Research Center for Biological Vector Control, Guangzhou, 510080 China

**Keywords:** Clonorchiasis, Obstructive jaundice, Gallstone

## Abstract

**Background:**

Hepatic clonorchiasis is one of the most prevalent food-borne parasitic diseases worldwide. *Clonorchis sinensis*, the pathogen, is the major parasitic trigger contributing to cholangitis, cholelithiasis, and even cholangiocarcinoma. Unfortunately, unspecific clinical manifestations of patients with hepatic clonorchiasis tend to mislead clinicians to neglect or misdiagnose them, following ignorance of appropriate therapy. Our case report may shed light on definite diagnosis of clonorchiasis with concomitant cholelithiasis, methodology for surgical drainage of the parasites, and postoperative anthelmintic therapy.

**Case presentation:**

Two patients with habit of eating infected raw or undercooked freshwater fish were hospitalized due to right upper quadrant pain and jaundice. Magnetic resonance cholangiopancreatography (MRCP)/computed tomography (CT) detection indicated cholangiolithiasis and cholangiolithiasis with concurrent cholecystolithiasis, respectively. Fecal examinations were both negative for adult worms or eggs of parasites. However, adults of *Clonrochis sinensis* were detected within hepatobiliary tracts during laparoscopic cholecystectomy. Postoperative drainage and anthelmintic therapy contributed to complete recovery with good prognosis.

**Conclusions:**

Clonorchiasis provokes cholangiolithiasis and cholecystolithiasis. Standardized treatments for these gallstone patients with concomitant clonorchiasis include surgical removal of the calculus, postoperative T tubule drainage and anthelmintic therapy. Serological test or polymerase chain reaction (PCR)-based approaches might be helpful for diagnosis of clonorchiasis when no eggs are found by stool microscopy. Public health promotion on ceasing to eat raw freshwater fish is essential for prevention and control of clonorchiasis.

## Background

Clonorchiasis, a serious foodborne zoonotic disese endemic in the Far East including China, Korea, Japan, and Vietnam, is caused by the pathogen *Clonorchis sinesis* (*C. sinensis*), which is mainly located within the intrahepatic ducts of patients. Humans can acquire the infection by ingesting raw or undercooked freshwater fish containing metacercariae [1–3]. However, the symptoms of hepatic clonorchiasis, including abdominal pain, nausea, anorexia, diarrhea, obstructive jaundice, and even pyogenic cholangitis, dispaly unspecific [4, 5]. Thus, it is hard to diagnose early until stool examination for eggs or worms harvested from the biliary tract during surgery. In this case report, we presented two cases of unspecific hepatobiliary manifestations of *C. sinensis* infection.

## Case presentation

### Case 1

**Fig. 1:**
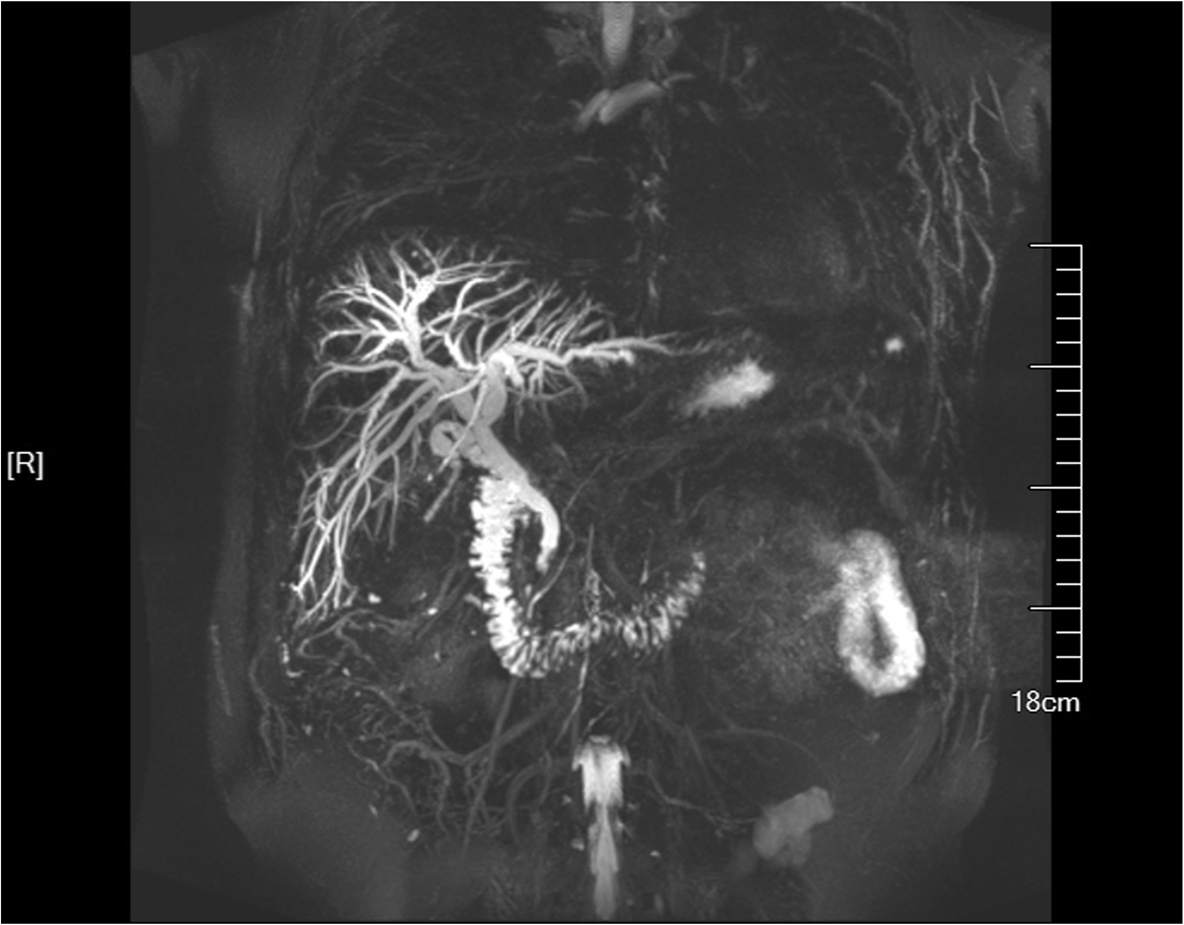
Dilation of the intrahepatic ducts by MRCP

**Fig. 2:**
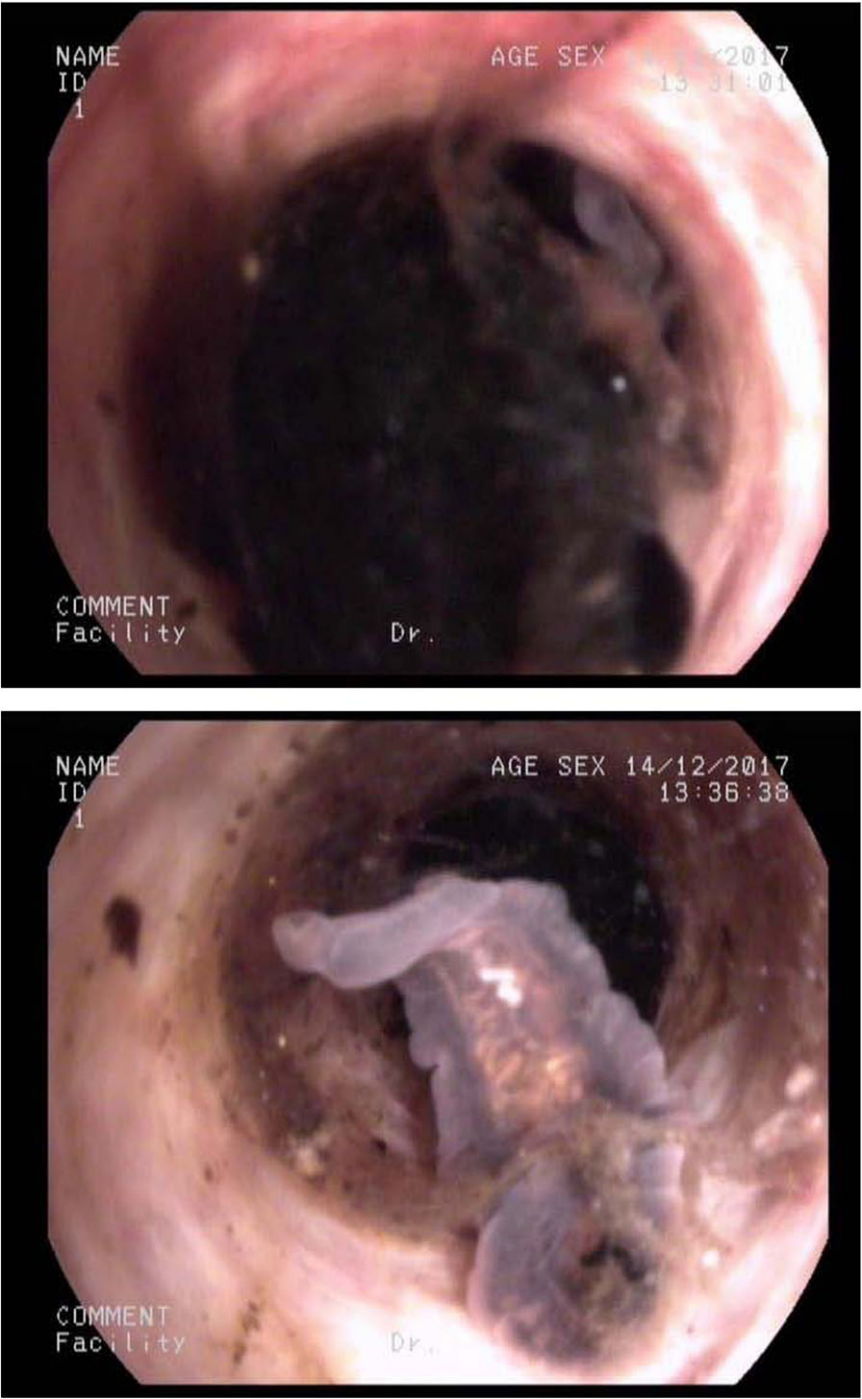
Examination of the stone and the worms in common bile duct by the choledochoscope during operation

A 49-year-old man complained of right upper quadrant abdominal pain and jaundice for 2 days and was hospitalized in the Fifth Affiliated Hospital of Sun Yat-sen University. The pulse rate of the patient was regular and the temperature and blood pressure were normal. The patient had a clear dietary history of eating raw freshwater fish, however, no eggs of parasites were detected in the stool specimen by direct smear method (each specimen was smear onto 3 labeled slides). Magnetic Resonance Cholangiopancreatography (MRCP) revealed obstruction of the common bile duct by a stone with obviously diffuse dilation of intrahepatic ducts (Fig. 1). Initial laboratory data indicated obstruction jaundice and liver enzymes elevation (Table 1). Considering that the cholangitis was caused by the common bile duct stone, the gallbladder stone and cholecystitis, The laparoscopic cholecystectomy and laparoscopic common bile duct exploration was performed. A flat, leaf-like worm was found under the choledochoscope at the extremitas inferior common bile duct during the operation (Fig. 2). After the surgery, a “T” shape catheter was inserted into the common hepatic duct to establish drainage. A course of anthelmintic therapy (albendazole: 16 mg/kg/day for 4 days) was administrated. During the subsequent days, the adult worms were observed in the bile duct through the “T” shape catheter. The pain of the patient relieved totally, the jaundice faded gradually and liver function indices were nearly normal.

**Table 1:** Pre-treatment blood results of 2 patients

Variable	Value		Reference value
	Patient 1	Patient 2	
Hematology			
Leukocytes	5.55 × 10^9/L	8.05 × 10^9/L	3.5–9.5 × 10^9/L
Neutrophil	59.60%	57.30%	40–75%
Lymphocyte	27.40%	20.70%	20–50%
Monocyte	6.50%	6.60%	3–10%
Eosinophils	6.50%	15.40%	0.4–8.0%
Basophils	0.00%	0.00%	0–1%
Erythrocytes	5.49 × 10^12/L	4.66 × 10^12/L	3.8–5.8 × 10^12/L
Hemoglobin	159 g/L	143.0 g/L	115-175 g/L
Hematocrit	47.10%	42.90%	35–50%
Platelets	190 × 10^9/L	230 × 10^9/L	125–350 × 10^9/L
Blood chemistry			
AST	83.00 U/L	182.00 U/L	13–40 U/L
ALT	236.00 U/L	399.00 U/L	7–50 U/L
γ-GTP	848.00 U/L	419.00 U/L	10–60 U/L
Total bilirubin	128.80 μmol/L	129.20 μmol/L	3-24 μmol/L
Direct bilirubin	92.20 mmol/L	96.90 mmol/L	0–8.0 mmol/L
ALP	315 U/L	315 U/L	45–125 U/L
TBA	233.00 μmol/L	242.90 μmol/L	0–10 μmol/L

Abbreviations: *ALT* alanine aminotransferase; *AST* aspartate aminotransferase; γ-GTP, gamma-glutamyl transpeptidase; *ALP* alkaline phosphatase; *TBA* total bile acid

### Case 2

**Fig. 3:**
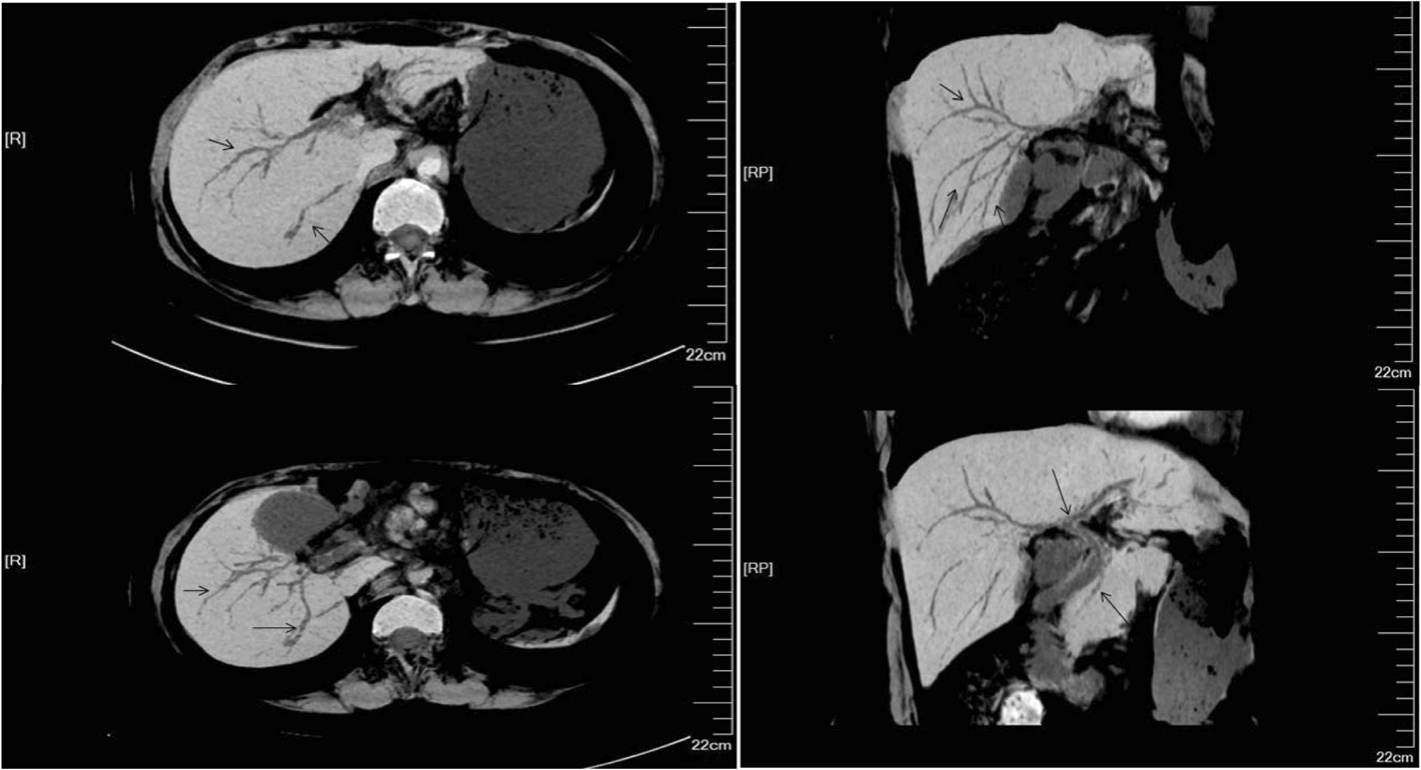
Dilation of the intrahepatic ducts by CT scan

**Fig. 4:**
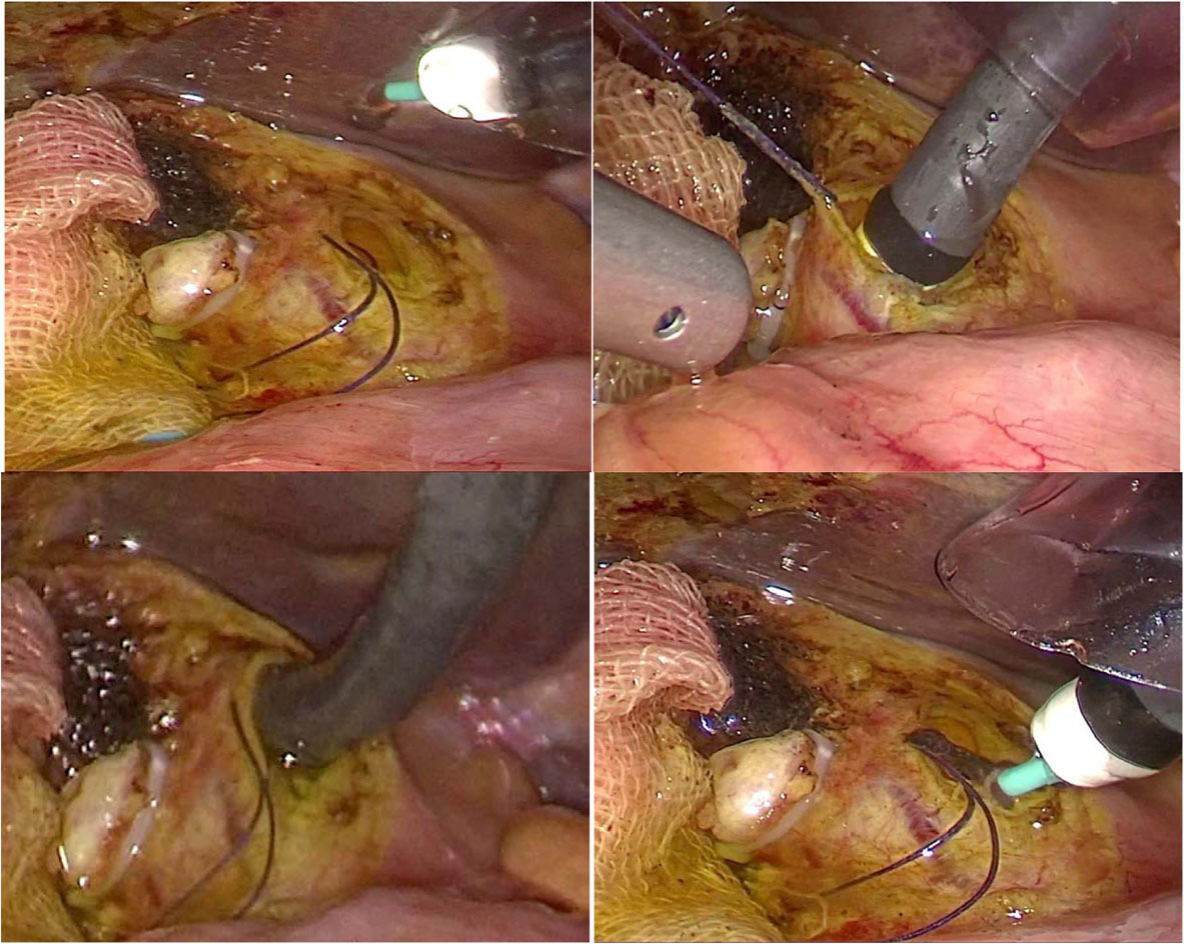
Laparoscopic view of the operation

**Fig. 5:**
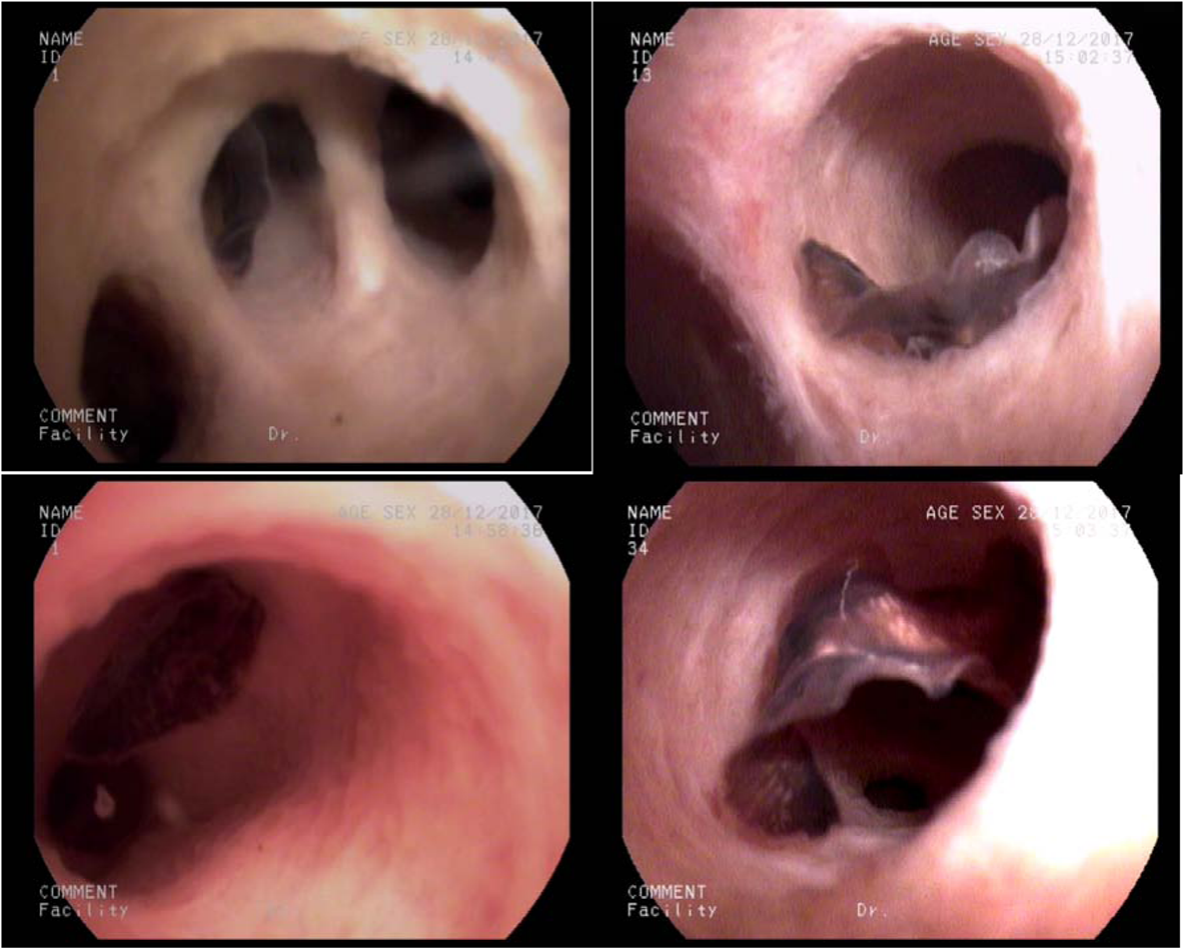
Choledochoscope view of worms inside the bile duct

**Fig. 6:**
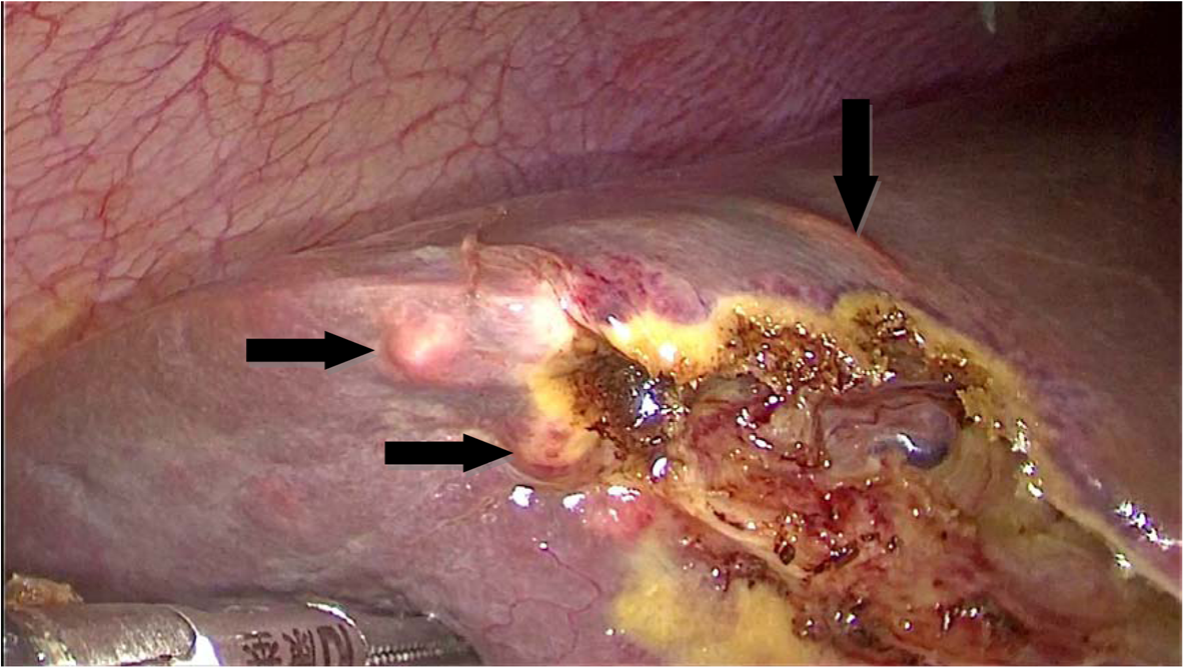
Nodules (indicated by arrows) distributed on the surface of the liver

**Fig. 7:**
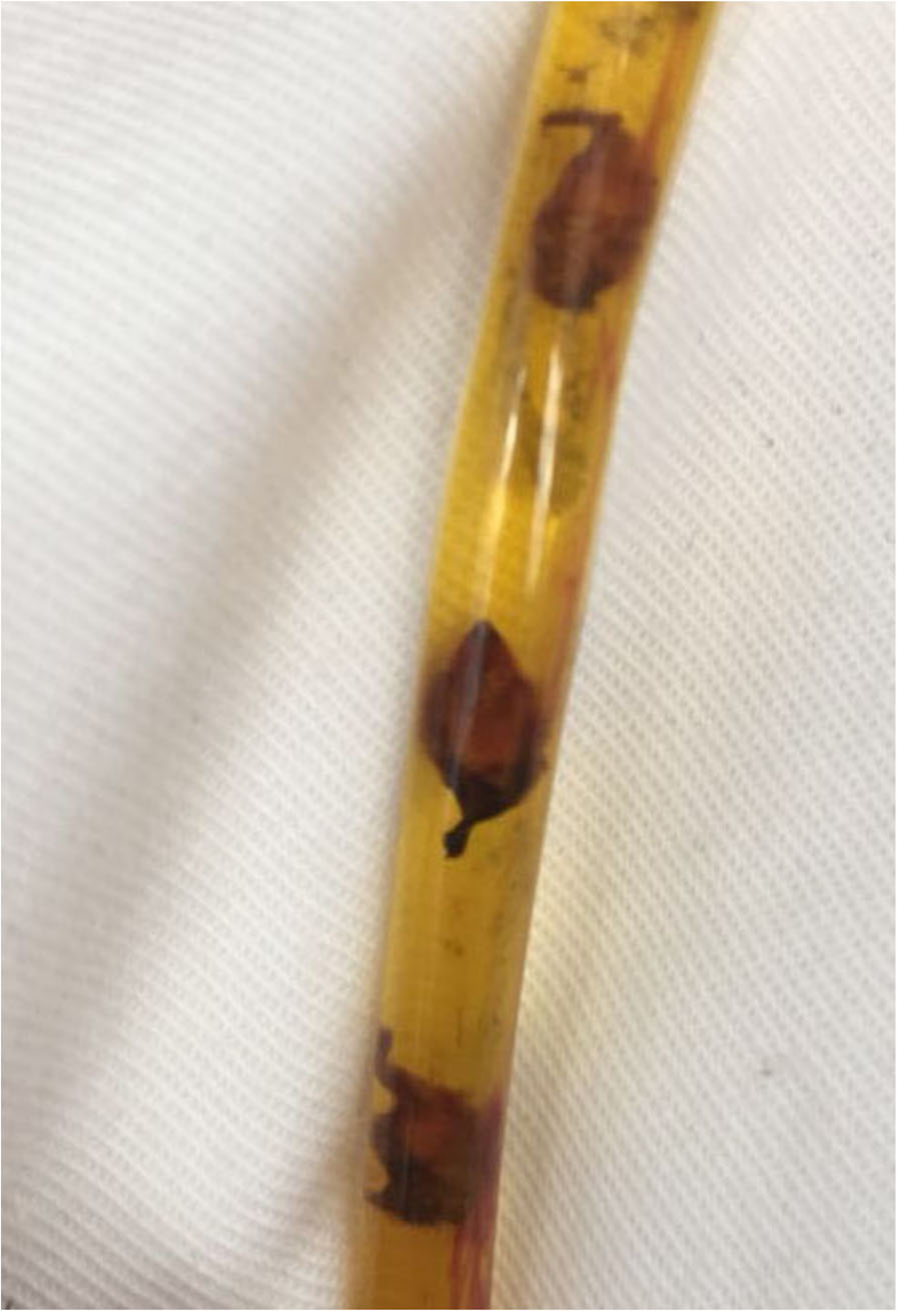
*C. sinensis* worms drained through the catheter

A 40-year-old female patient had suffered from upper quadrant abdominal pain, with occasional nausea and fever for more than 2 years. The patient was diagnosed as cholecystitis at another hospital nearby and felt relieved very soon after treatment in the past 2 years. She came to the hospital 11 days ago due to the severe upper quadrant abdominal pain recurred with severe jaundice. Complete medical examinations, including blood pressure, pulse rate, temperature and physical examination of the abdomen, were performed in the Fifth Affiliated Hospital of Sun Yat-sen University. The clinical examinations revealed that the pulse rate, temperature and blood pressure were normal. Stool microscopy for parasite eggs by direct smear method were negative 3 times. Computed tomography (CT) scan revealed obstruction of the bile duct with dilation of the intrahepatic ducts which suggested a retained bile duct stone and a gallbladder stone (Fig. 3). Laboratory data indicated obstruction jaundice, peripheral eosinophilia and liver enzymes elevation (Table 1). Detailed inquiry revealed she had a history of eating raw freshwater fish. A clinical diagnosis of acute cholangitis and cholecystitis was made and laparoscopic cholecystectomy and laparoscopic common bile duct exploration was performed. Many flat, leaf-like worms appeared under the choledochoscope as deep bile duct cannulation (Fig. 4 and Fig. 5). Besides, many nodules distributed dispersedly among the surface of the liver (Fig. 6). After the operation, the“T” shape catheter was placed in the common hepatic duct to allow patent drainage. The patient was treated with anthelmintic therapy (albendazole: 16 mg/kg/day for 4 days). More *C. sinensis* worms were drained through the “T” shape catheter (Fig. 7). The clinical status of the patient improved gradually without the pain recurring.

## Discussion and conclusions

Clonorchiasis is the most common fluke infection in East Asian population with approximately 35 million infected individuals [6, 7]. The encysted larvae of the parasites invade the hosts mostly by the consumption of raw freshwater fish and crustaceans, release in the duodenum, migrate into the bile ducts and develop into adults within the host’s intrahepatic ducts [8]. *C. sinensis* infection is primarily related to liver and biliary tract disorders and considered as the etiological agents of cholangiocarcinoma [9–11]. However, the clinical manifestations of hepatic clonorchiasis are usually unspecific, including nausea, anorexia, diarrhea (with moderate infestation), right upper quadrant abdominal pain, obstructive jaundice, tender hepatomegaly and pyogenic cholangitis (with severe infestation) and mild infestation is asymptomatic [12–14], so it’s easy to be neglected or misdiagnosed.

Some incidental diagnosis is suspected due to discovery of ova on routine stool examination. Although our patients had the history of eating inadequate cooked freshwater fish, the negative results of stool examination for eggs in the present study might resulted from the low rates of egg detection in the feces [15], and determination of *C. sinensis* DNA in feces by real-time fluorescent PCR [16] or serological test for *C. sinensis* - specific antibodies will increase the sensitivity and hence contributes to the diagnosis [17, 18] .

Administration of anthelmintic medication, albendazole (an effective alternative of praziquantel), is the primary treatment for clonorchiasis [19, 20]. Meanwhile, when there is biliary obstruction caused by the worm directly or indirectly, the operation is necessary. In the present study, both of the two patients had acute cholangitis with presentation of pain and obstructive jaundice which most likely resulted from the bile duct obstruction by a mass of adults of *C. sinensis* [21]. The worms obstructed the biliary tree and served as a nidus for biliary tract stones formation. Therefore, presence of biliary tract stones or bile duct obstruction cannot exclude the possibility of clonorchiasis [22]. Moreover, Long-term infection may result in chronic inflammation and adenomatous hyperplasia of the bile duct which may be a potential risk factor for cholangiocarcinoma [23, 24]. In the second case, we found numerous nodules which differed from cirrhotic nodules distributed dispersedly on the liver surface during the operation. On examination the nodules had the following characteristics: waxy, immobile, larger volume and the texture equal to the liver. An incisional biopsy of the nodule was performed and the histopathological findings of the excised lesion were consistent with those of the dilated biliary duct cysts caused by clonorchiasis [25]. It reminds the surgeons if similar liver nodules were discovered during the operation, Hepatic clonorchiasis should be considered consciously as one of the possibilities, especially the patients have the history of eating raw freshwater fish. Due to the limited diagnostic value of CT, further investigation points to MRCP, which can clearly outline the caliber, direction and expansion of intrahepatic bile ducts involved [14]. More precise presentation includes minor “cystic” dilatation of peripheral branches of bile ducts, which is quite different from the “homogeneous” dilatation of bile ducts caused by gallstones or tumors. But still it is not significant enough for radiologists to find out the exact pathogen, either the adult worm or the eggs [23].

In summary, the clinical symptoms of patients infected by *C. sinensis* are protean. The diagnosis should be suspected when there are following manifestations: recurrent cholangitis, obstructive jaundice, dilation of the intrahepatic ducts without extrahepatic obstruction by CT scan or MRI [26], peripheral eosinophilia, and consumption history of raw freshwater fish. The most effective means to control the disease is to educate people and advertise avoiding eating raw freshwater fish, perhaps [27]. There lacks specific clinical manifestations indicating the accurate diagnosis of hepatic clonorchiasis, partially illustrating why clinicians tend to neglect or misdiagnose it especially as comorbidity in most cases. In a proper epidemiologic context, presence of gallstones and bile duct obstruction can be a sign of clonorchiasis, because even in assymptomatic patients, some of the most common findings in ultrasonography are gallstones and sludge [13, 21].

## Abbreviations

CT: Computed Tomography
MRCP: Magnetic Resonance Cholangiopancreatography
MRI: Magnetic Resonance Imaging
